# Socioeconomic and cultural factors associated with pap smear screening among French women living in Réunion Island

**DOI:** 10.1186/s12889-024-18633-4

**Published:** 2024-04-23

**Authors:** Rémi Houpert, Marc-Karim Bendiane, Laetitia Huiart, Anne-Deborah Bouhnik, Caroline Alleaume, Rajae Touzani, Jacqueline Veronique-Baudin, Julien Mancini, Clarisse Joachim, Emmanuel Chirpaz

**Affiliations:** 1 Aix Marseille Univ, Inserm, IRD, SESSTIM, Sciences Economiques & Sociales de la Santé & Traitement de l’Information Médicale, ISSPAM, Marseille, France; 2 Research & Development in Oncology (UF3596), Oncology Hematology Urology department, University Hospital of Martinique, Fort-de-France Martinique, Martinique; 3 Internal Medicine, Geriatrics and Therapeutic Unit, AP-HM, Marseille, France; 4 Santé Publique France, Paris, France; 5 APHM, Hop Timone, BioSTIC, Biostatistique et Technologies de l’Information et de la Communication, Marseille, France; 6 General Cancer Registry (UF 1441), Oncology Hematology Urology department, University Hospital of Martinique, Fort-de-France Martinique, Martinique; 7 Reunion cancer Registry - Clinical Investigation Center (INSERM CIC-1410), University Hospital FR, Saint Pierre Cedex, France

**Keywords:** Cervical cancer, Pap smear screening, Reunion Island, Health behaviors

## Abstract

**Background:**

Réunion Island is a French overseas territory located in the southern Indian Ocean, with a challenging socioeconomic and multicultural context. Compared to mainland France, Réunion has an overincidence and overmortality of cervical cancer. In order to investigate these two issues, it is important to evaluate the barriers and potential levers to Pap smear screening among female inhabitants of the island. We aimed to identify the specific socio-demographic factors, cultural factors, and living conditions associated with Pap smear screening in Réunion, with a view to increasing uptake.

**Methods:**

We conducted a Knowledge Attitude Behavior and Practices (KABP) survey on cervical cancer screening practices among women aged between 25 and 65 years old living in Réunion Island, selected using random digit dialing sampling. Data were collected using Computer Assistant Telephone Interviews. Weighted chi-squared tests and Student’s t-tests were used to compare women who had up-to-date Pap smear screening with women who did not. Weighted logistic models were used to identify the factors associated with not having up-to-date screening.

**Results:**

A total of 1000 women were included in the study. Of these, 88.1% had a Pap smear test during the previous three years. Factors independently associated with not being up to date were as follows: aged over 55 (AOR 2.3 [1.2–4.3]), no children (AOR 2.5 [1.4–4.3]), having free universal health coverage (AOR 1.7 [1.1–2.7]), an income per unit consumption lower than 1500€ per month (AOR 2.0 [1.1–3.7]), low health literacy (AOR 2.7 [1.7–4.1]), not consulting a general practitioner in the prior 12 months (AOR 3.6 [2.0-6.5]), and a BMI > 30 (AOR 2.6 [1.5–4.4]).

**Conclusions:**

This is the first large-scale survey focusing on recommended Pap smear screening uptake in Réunion Island. Although self-reported screening incidence was higher than in mainland France, national screening policies must take into account the island’s diverse social and cultural characteristics (e.g., an ageing population, low health literacy), while implementing actions to fight against poverty and increase general access to healthcare.

**Supplementary Information:**

The online version contains supplementary material available at 10.1186/s12889-024-18633-4.

## Background

In France, of the approximately 3,000 women diagnosed with cervical cancer (CC) each year, 1,000 die from it. CC is the 12th leading cause of death by cancer among women in the country. Réunion Island (Réunion hereafter) is a French overseas administrative territory located in the southern Indian Ocean (Supplementary file 1). There is full continuity in state policies and services between the mainland and the island. CC is the 4th most common cancer among women in Réunion, and there is a significant overincidence compared with mainland France [1]. Specifically, the age-standardized incidence rate of CC in Réunion in 2017 was 9.7 per 100,000 person-years, compared with 6.1 in 2018 in mainland France. Moreover, this rate was 4.4 per 100,000 person-years in 2013–2015, double that observed in mainland France [2, 3].

The population of Réunion (850,727 inhabitants in January 2023) is relatively young for a developed country (30% are aged under 20 years) and is very cosmopolitan. There are six main ethnic groups and four main religions (Islam, Christianity, Buddhism, and Hinduism), the consequence of European, African, Malagasy, Indian and Chinese migrations over the past 350 years. The island has some of the poorest socioeconomic indicators in France, with an unemployment rate estimated at 17% in 2022, and 36% of the population living below the French metropolitan poverty threshold in 2020. In addition, due to its mountainous geography and the lack of public transport, some rural populations are isolated and have limited access to healthcare services. Consequently, socioeconomic disparities are particularly significant, and are largely responsible for social and territorial disparities in terms of health. These disparities can be seen in the levels of personal exposure to health risk factors, including genetic components [4], health behaviors [5], cancer experience [6] and individual screening strategies [7].

Despite the recent introduction of vaccination against human papillomavirus (HPV), Pap smear screening remains the only reliable means of cervical cancer prevention. It has greatly helped to reduce cervical cancer incidence and mortality, particularly in developed countries [8, 9]. In Réunion, vaccination against HPV began in 2007 and is aimed at people aged 15–18. Vaccination coverage was estimated at 24% [10] and screening coverage was estimated at 60% in 2017 in Réunion [11], far from the national target of 81.4% for screening [12].

For many years, public health interventions have been implemented in French mainland and overseas territories by successive governments to provide access to facilities offering adequate cancer prevention and effective treatment [13]. One example is the CC screening program; initiated in 2018, its aim is to complement spontaneous screening services by inviting women aged 25 to 65 years old who are not up-to-date with their Pap smear screening, to perform either a Pap test or a HPV test every 3 to 5 years, depending on their age and most recent screening result. However, results to date for this program have been underwhelming, given the resources allocated (including the mobilization of local actors). Coverage rates are still low, and the continued declining trend in Pap smear screening uptake is a growing public health issue [12]. 

Many barriers to Pap smear screening have been documented in the literature, including socio-demographic factors, health behaviors, health status, poor knowledge of CC and its treatment, socio-cultural factors, and equipment limitations [14–16].

To date, only one qualitative anthropological study, conducted in 2002, has focused on Pap smear screening uptake in Réunion. It found two attitudes which were age dependent. Specifically, women under 50 years old (a sub-population with a better understanding of their sexuality and femininity) were more receptive to screening, while older women (who had more traditional psycho-sociological values) felt less concerned by the disease, were more open to the idea of mortality, are were less inclined to go for screening [17].

Considering the particular context of Réunion, as discussed in the previous paragraphs, it is important to identify the specific socio-demographic factors, cultural factors, and living conditions associated with Pap smear screening, with a view to increasing uptake. Given the many native ethnic and religious minorities in this local insular population, we hypothesize that it has a specific social organization which is based on the historic relationships between these minorities [18]. Accordingly, we aimed to determine the relative contribution of different kinds of social and cultural factors to CC screening behaviors of the women living in Réunion, taking into account not only ethnicity but also religion [19]. This latter element comprises a new challenge in epidemiology in the French context.

## Methods

### Study design

FOSFORE is a Knowledge Attitude Behavior Practices (KABP) survey on CC screening practices which was conducted in 2017 among women living in Reunion Island [20]. Data were collected between March and June 2017 using telephone interviews among a sample of residents, aged between 25 and 65 years. Eligibility criteria were being able to speak French or Creole, having the physical and cognitive capacity to answer a telephone-based questionnaire, and no history of hysterectomy or uterine conization. Given that a very large majority of households in Réunion only have mobile phones (84% in 2023) [21], we constituted our random sample based on the following two sampling frames [22]: (i) households subscribing to a land-line operator, with or without a mobile line, and (ii) women who only had a mobile phone number. When contacting land-line numbers, random selection among all eligible women in the household was performed using the usual Random Digit Dialing (RDD sampling rules [23].

We calculated that a study sample of 1000 women was required [24, 25]. Details of the sample calculation are explained in the Supplementary file 2.

### Measures

The telephone interviews included between 75 and 150 questions relating to socio-demographic characteristics, living conditions, health, utilization of health services, disease prevention practices, religious beliefs, knowledge and beliefs about CC, and attitudes towards Pap smear screening. Most were closed questions with response scales or multiple-choice answers.

#### Up-to-date pap smear screening

As part of the health-based questions, women were asked if they had ever had a Pap smear screening (yes / no / does not know). Those who answered “yes” then indicated how long it had been since their last test (less than 1 year / between 1 and 2 years / between 2 and 3 years / between 3 and 5 years / more than 5 years / does not know). In accordance with French guidelines [26], persons who reported having a test in the previous 3 years were considered to be up to date with their screening. All others were considered not to be up to date.

#### Health literacy

Health literacy was measured using the validated French-language version of the third scale (‘Actively Managing my Health’) of the Health Literacy Questionnaire (HLQ) [27]. This scale comprises five items scored on a four-point Likert-type response scale (strongly disagree, disagree, agree, strongly agree). A mean score between 1 and 4 was calculated. A score lower than 3 was considered to represent low health literacy.

#### Income per unit of consumption

Self-reported income was collected which included all household resources. Income per unit of consumption was calculated by dividing household resources by household weight, calculated as follows: the value 1 for the head of the household, 0.5 for each of the other persons aged 14 years or older, and 0.3 for each child aged under 14 [28]. Households with an income per unit of consumption below 1500€ were considered to have low incomes.

#### Frequency of General Practitioner (GP) consultations

Women were asked how many times they had consulted a GP in the preceding 12 months.

#### Free universal health cover

At the time of this study, several different health allowances existed in France for people on low income. These included complementary health solidarity such as Complementary Universal Coverage (*Couverture Maladie Universelle Complémentaire* or CMUC), assistance with paying health care mutuals (*Aide Complémentaire de Santé* or ACS), and state medical aid for irregular non-French nationals (*Aide Médicale d’état* or AME). Participants who received at least one of these allowances were defined as receiving free universal health cover.

### Statistical analyses

A weighting procedure was applied to ensure that the data were representative of the population of Réunion in relation to age and professional activity. Weighted chi-squared tests and Student’s t-tests were used to compare women up to date with their Pap smear screening with those who were not. Weighted logistic models were then used to identify factors associated with not being up to date, A step-by-step procedure was performed to select the statistically significant factors to retain in the multivariate model. The significance thresholds were 20% and 5%, respectively, in the univariate and multivariate analyses.

## Results

16,711 women were contacted by phone over the four months of data collection, and 7,186 were eligible to participate. Among them, 1,184 agreed to participate in the survey and 184 stopped before the end of completion. Finally, one thousand women were included in the study. Mean age was 43.7 years (SD = 11.1), 49.5% had a level of education below upper secondary school certificate, 65.9% were professionally active at the time of the survey, 62.0% had a low income, 60.4% were living with a partner, and 13.1% had no child (Table 1).

**Table 1:** Factors associated with being up to date with Pap smear test or not – univariate analyses – (*n* = 1000)

	Total %	Up to date with Pap smear test		*p* ^a^
		YES 88.1%	NO 11.9%	
**Age (years)**				0.038
25-35	27.5	27.4	28.6	
36-45	28.0	29.2	19.3	
46-55	27.1	27.1	26.9	
56-65	17.4	16.3	25.2	
**Level of education**				0.105
No diploma	29.5	28.0	39.5	
< high school certificate	20.0	20.6	16.8	
high school certificate	18.7	19.3	13.4	
> high school certificate	30.4	31.0	26.9	
Missing	1.4	1.1	3.4	
**Religion**				0.220
Christian	69.7	69.9	68.1	
Muslim	3.5	3.1	6.7	
Hindu	4.8	5.1	2.5	
Other	2.7	2.6	3.4	
None	19.2	19.2	19.3	
Missing	0.1	0.1	-	
**Living as a couple**				0.006
No	39.6	38.0	51.3	
Yes	60.4	62.0	48.7	
**Children**				<.001
None	13.1	11.8	22.7	
1 or 2	51.8	53.6	38.6	
3 or more	35.1	34.6	38.7	
**Professional situation**				<0.114
Active	65.9	67.0	57.6	
Inactive	27.4	26.5	33.9	
Retired	6.4	6.1	8.5	
Missing	0.3	0.4	-	
**Income per consumption unit (€)**				<.001
< 1500	62.0	60.2	75.4	
≥ 1500	29.8	31.7	16.1	
Missing	8.2	8.1	8.5	
**Free universal health coverage**				0.003
No	70.6	72.2	58.8	
Yes	29.1	27.6	40.3	
Missing	0.3	0.2	0.9	
**Adequate health literacy**				<.001
No	30.7	28.5	47.1	
Yes	65.9	68.2	48.7	
Missing	3.4	3.3	4.2	
**Consulted a GP at least once in the previous 12 months**				<.001
No	7.6	6.2	17.6	
Yes	92.4	93.8	82.4	
**Consulted a dentist at least once in the previous 12 months**				0.028
No	39.4	38.1	51.3	
Yes	60.3	61.5	48.7	
Missing	0.3	0.4	-	
**Consulted a gynaecologist at least once in the previous 12 months**				<.001
No	51.9	46.7	90.8	
Yes	47.5	52.7	9.2	
Missing	0.6	0.6	-	
**Up to date with mammography screening**				<.001
No	20.9	9.1	32.8	
Yes	11.9	22.2	10.9	
Not concerned (age<50)	67.2	68.7	56.3	
**BMI**				<.001
Underweight	4.6	4.5	5.0	
Normal	51.4	53.0	40.4	
Overweight	27.8	28.1	25.2	
Obese	16.2	14.4	29.4	
**Already heard about HPV vaccine**				<.001
No	36.5	33.9	55.5	
Yes	63.0	65.9	42.0	
Missing	0.5	0.2	2.5	

^a^calculated excluding missing values

Slightly under a third (29.1%) reported having access to free universal health cover, while 92.4%, 60.3%, and 47.5% reported at least one consultation with their GP, dentist, and gynecologist in the previous twelve months, respectively. Overall, 30.7% had low health literacy (mean (SD) score for Scale 3 of the HLQ = 3.0 (0.6)). Most (80.7%) of the participants had a religion, the majority of whom were Christian (69.7%).

Almost all the participants (96.2%) had already had a Pap smear test during their lifetime. Of these, 88.1% were up to date while 8.1% were not.

Table 1 present the univariate analyses of the factors associated with not being up to date.

Women not up to date were more likely to be aged over 55 years (25.2% versus 16.3% *p* = 0.038), but less likely to be aged between 36 and 45 years (19.3% versus 29.2%, *p* = 0.038).

Moreover, not living with a partner, not having children, having health literacy difficulties, an income per unit consumption beneath 1500€, and access to free universal health cover, were all associated with a higher likelihood of not being up to date.

In contrast, consulting a GP, a dentist or gynecologist in the 12 months prior to the survey, having heard about HPV vaccine, having a normal BMI, and - for women over 50 years - being up to date with recommended mammogram screening, were all associated with a higher likelihood of being up to date.

Finally, no relationship was found between Pap smear screening and religious beliefs or with education level.

After multiple adjustment, the factors associated with not being up to date with Pap smear screening were as follows: over 55 years, childless, receiving free universal health cover, having a low income, having low health literacy, not consulting a GP in the previous 12 months, and having a BMI > 30 (Table 2).

**Table 2:** Factors associated with not being up to date with Pap smear test – multivariable analyses – (*n* = 961*^a^)

	Adjusted OR	CI (95%)	*p*
**Age classes (years)**			0.003
25-35	1		
36-45	0.68	0.36-1.26	
46-55	1.37	0.77-2.42	
56-65	2.31	1.23-4.31	
**Children**			0.002
None	2.46	1.41-4.30	
1 or more	1		
**Free universal health coverage**			0.023
No	1		
Yes	1.71	1.08-2.71	
**Income per consumption unit (€)**			0.047
< 1500	2.05	1.14-3.67	
≥ 1500	1		
Missing	2.18	0.91-5.24	
**Health literacy difficulties**			<.001
Yes	2.68	1.74-4.12	
No	1		
**Consulted a GP at least once in the previous 12 months**			<.001
No	3.60	1.99-6.51	
Yes	1		
**BMI**			0.003
Underweight	1.32	0.50-3.49	
Normal	1		
Overweight	1.07	0.63-1.80	
Obese	2.57	1.51-4.37	

^a^ *n* = 961, total sample calculated excluding missing values

## Discussion

This is the first large-scale survey focusing on recommended Pap smear screening in Réunion. Despite random sampling, one of the major limitations was the lack of representativeness of some religious minorities such as Muslims and Hindus, and vulnerable hard-to-reach sub-groups including migrants, very low-income families, and non-French/Creole speaking people (Supplementary file 3). Other limitations mirror those usually associated with RDD household surveys. Specifically, questions on sensitive issues, like sexual relations, often generate inexact estimations because of social desirability bias [29]. Accordingly, as Pap smear screening was self-reported, the assessed coverage rate (88.1% vs. 60% in previous survey) may have been overstated because of this bias. Despite these limitations, the study design ensured that results could be compared at the national and international levels. To ensure sub-national and international comparability of cancer-related health behaviors in France, the “Baromètre Cancer” cross-sectional telephone survey was introduced by the second national cancer plan (2009–2013) and definitively incorporated into government cancer strategies through the third cancer plan (2014–2019) [30, 31]. FOSFORE, which focuses on CC prevention practices, has therefore followed the design rules of the “Baromètre Cancer” to ensure the comparability and external validation of its results. Consequently, they can provide specific comparison-based information on individual and social factors associated with screening behaviors among in this overseas population.

Social inequality is a major determinant of population health [32–34], and many studies have reported that socioeconomic status (SES) has a significant impact not only on cancer diagnosis, treatment and mortality [35–37], but also on cancer prevention, including screening behaviors and practices. In high-income countries, low screening coverage is related to current health and social inequalities [38]. Our results on the association between SES and CC screening behaviors reflect the literature [14, 39, 40]. Specifically, we found that SES disparities were more strongly associated with Pap smear screening uptake in Réunion than in mainland France, irrespective of culture and religion. However, we did not find any significant relationship between culture or religion. This would suggest that in order to improve screening we must first and foremost consider a woman’s SES (which we measured by two proxies in the present study: receiving free universal health coverage (or not) and low income (or not)).

Moreover, despite comprehensive national (i.e., covering the mainland and overseas territories) policies on access to health care in France, real-world access for women living in overseas territories is more difficult due to a high level of social vulnerability (higher unemployment rates, higher cost of living, etc.) [41, 42]. Given the relatively low uptake of Pap smear screening by socioeconomically vulnerable women in Réunion, the French health care system must promote tailored interventions able to respond to this population’s specific needs [43].

In our study, women over 55 years old were significantly less likely to be up to date with their Pap smear screening, which is consistent with the most recent French national observational survey on cancer [12]. There are several factors which may explain this finding, including an ever-decreasing number of gynecological consultations [44], and fewer problems associated with menstrual disorders [45]. Our findings highlight the importance for all healthcare providers - in particular GP - to pay more attention to women over 55 years old, given their higher risk of CC [46].

In line with previous studies, our multivariate analysis showed that regularly consulting a GP could play a major role in improving Pap smear screening uptake [47]. However, a recent French study concluded that the absence of regular consultations with a healthcare practitioner (including GP) cannot fully explain why some women do not go for screening [48]. Only implementing interventions to solve problems associated with medical demography (e.g., a lack of GP and of medical staff.) is certainly not sufficient to guarantee optimal screening coverage.

Several individual factors, including empowerment and health literacy, can play a role in women’s uptake of Pap smear screening. Empowerment is essential in this context, as shown by the negative impact of difficulties in actively managing one’s health (see scale 3 of the HLQ) in our study. Health literacy is not only about accessing and understanding health information; it involves motivation and the competency to appraise and apply information for decision-making [49]. Most studies in the last decade have focused on lower income and level of education as barriers to Pap smear screening (and cancer screening in general) without investigating the influence of low health literacy [50]. Long-term plans to increase all domains of health literacy in the general population and specific interventions to help women with low literacy could lead to increased screening uptake. Furthermore, national screening campaigns must be adapted to local populations in terms of their level of health literacy. As one might have expected given the context [51], the level of health literacy (mean = 3.0 for scale 3 of the HLQ) in our survey was lower than that recorded in the general Australian population (mean = 3.09, mean in the first quintile of equivalized income of household = 3.02) [52]. This level was also lower than that in a small sample of citizens from a deprived borough in mainland France (mean = 3.2) [53].

To understand the role played by women’s weight in Pap smear screening uptake, a systematic review exploring attitudes toward preventive behaviors showed that women with overweight are less likely to go for screening. The authors suggested that poor body self-image and negative experiences during gynecological examinations were partly to blame for this [54], and underlined the need for professionals to take into account possible patient embarrassment. These findings are consistent with our survey, where women with a high BMI in Reunion are less likely to be up to date with their smear tests. These findings were also confirmed by a French study on cervical cancer screening among women living in overseas territories [11, 55], where obesity was a socio-demographic factor significantly associated with non-completion of recent screening. To increase Pap smear screening uptake by this population, targeted interventions are needed.

Multivariate analysis showed that having children was positively associated with Pap smear screening in our study. Previous published research on adult daily life habits, such as tobacco smoking, concluded that having children is associated with better disease prevention health behaviors [56]. This could mean that women with parental responsibilities are more attentive to their own health.

Finally, our study comes after 10 years of awareness-raising on the HPV vaccination recommendation in France and in Réunion for girls aged 11 to 14 (started in 2007), and highlights better Pap smear screening coverage in Réunion (88.1% vs. 76.8%) than in metropolitan France [12]. This coverage rate is overestimated due to social desirability and lack of representativeness. In these 10 years, the national recommendation has sufficiently covered the women who responded to our survey and could not change the results obtained. Nevertheless, there is still an over-incidence of CC (and an incidence of CC-related mortality) on the island, just as is the case in other French overseas territories. In addition, despite the effectiveness of the vaccine, HPV vaccination coverage in Réunion, as in mainland France, remains woefully inadequate. Around 14% of girls on Reunion Island, compared with 41.5% in mainland France [10, 11]. It is therefore essential to acquire a much greater understanding of the continued reluctance of some women in these areas to go for screening.

## Conclusions

The diversity of factors influencing health behaviors, and in particular screening practices among women, make it difficult to adequately implement health interventions to improve screening uptake, both in mainland France and the country’s overseas territories, including Réunion. In terms of the latter territory, national policies to increase Pap smear screening uptake must take into account the island’s diverse social and cultural characteristics (e.g., an ageing population, low health literacy), while implementing actions to fight against poverty and increase general access to healthcare.

### Supplementary Information

**Supplementary Material 1.**

**Supplementary Material 2.**

**Supplementary Material 3.**

## Data Availability

The datasets generated and/or analyzed during the current study are not publicly available, but can be made available from the corresponding author on reasonable request.

## Abbreviations

AME: Aide Médicale d’Etat
BMI: Body Mass Index
CC: Cervical Cancer
FOSFORE: Freins et leviers au dépiStage par Frottis cervico-utérin du cancer du cOl de l’utérus à la Reunion
GP: General Practitioner
HLQ: Health Literacy Questionnaire
HPV: Human Papilloma Virus
KABP: Knowledge, Attitudes, Behaviors and Practices
RDD: Random Digit Dialing
SD: Standard deviation
SES: Socioeconomic Status

## References

[1] Defossez G, Le Guyader-Peyrou S, Uhry Z, Grosclaude P, Colonna M, Dantony E, et al. *Estimations nationales de l’incidence et de la mortalité par cancer en France métropolitaine entre 1990 et 2018. Volume 1 – Tumeurs solides.* Saint-Maurice (Fra) : Santé publique France, 2019. 372 p. Available from: http://www.santepubliquefrance.fr/; https://geodes.santepubliquefrance.fr; http://lesdonnees.e-cancer.fr/; https://www.e-cancer.fr/

[2] Les Cancers à La Réunion. Observatoire Régional de la Santé Océan Indien; 2019. http://registre-cancer.re/wp-content/uploads/2021/08/tdb_cancers_reunion_2019.pdf.

[3] Données d’incidence pour les principales localisations tumorales chez les femmes: année. 2017. Registre des Cancers de la Réunion. http://registre-cancer.re/donnees-reunion-femmes/.

[4] Beavis AL, Gravitt PE, Rositch AF (2017). Hysterectomy-corrected cervical cancer mortality rates reveal a larger racial disparity in the United States. Cancer.

[5] Saulle R, Sinopoli A, De Paula Baer A, Mannocci A, Marino M, De Belvis AG (2020). The PRECEDE-PROCEED model as a tool in public health screening: a systematic review. Clin Ter.

[6] Schaafsma J, Nezlek JB, Krejtz I, Safron M (2010). Ethnocultural identification and naturally occurring interethnic social interactions: Muslim minorities in Europe. Eur J Soc Psychol.

[7] Smith RA, Andrews KS, Brooks D, Fedewa SA, Manassaram-Baptiste D, Saslow D (2017). Cancer screening in the United States, 2017: a review of current American Cancer Society guidelines and current issues in cancer screening. CA Cancer J Clin.

[8] Mishra R, Bisht D, Gupta M (2022). Primary screening of cervical cancer by pap smear in women of reproductive age group. J Family Med Prim Care.

[9] Woronoff AS, Molinie F, Tretarre B (2019). [Implementation of national cervical cancer screening program in France]. Bull Cancer.

[10] Hanguehard Rémi GA, Soullier N, Anne-Sophie B, du Chatelet P, Isabelle VS (2022). Couverture vaccinale contre les infections à papillomavirus humain des filles âgées de 15 à 18 ans et déterminants de vaccination, France. Bullet épidémiologique hebdomadaire.

[11] Hamers FF, Jezeweski-Serra D. Couverture Du dépistage Du cancer du col de l’utérus en France, 2012–2017. Bull Epidémiol Hebd. 2019;(22–3):417–23. http://beh.santepubliquefrance.fr/beh/2019/22-23/2019_22-23_2.html.

[12] ©Baromètre cancer 2021 / Institut national du cancer et Santé publique France, janvier 2023. https://www.santepubliquefrance.fr/determinants-de-sante/tabac/documents/rapport-synthese/barometre-cancer-2021.-attitudes-et-comportements-des-francais-face-au-cancer

[13] INCA. Le Plan cancer 2014–2019. Available from: https://www.e-cancer.fr/Institut-national-du-cancer/Strategie-de-lutte-contre-les-cancers-en-France/Les-Plans-cancer/Le-Plan-cancer-2014-2019.

[14] Broberg G, Wang J, Ostberg AL, Adolfsson A, Nemes S, Sparen P (2018). Socio-economic and demographic determinants affecting participation in the Swedish cervical screening program: a population-based case-control study. PLoS One.

[15] Chorley AJ, Marlow LA, Forster AS, Haddrell JB, Waller J (2017). Experiences of cervical screening and barriers to participation in the context of an organised programme: a systematic review and thematic synthesis. Psychooncology.

[16] Oldach BR, Katz ML (2014). Health literacy and cancer screening: a systematic review. Patient Educ Couns.

[17] Durolek D. Approche anthropologique des réticences des femmes face au frottis à l’île de la Réunion. Thèse de doctorat en Médecine, Faculté de médecine de Dijon; 2002.

[18] Jean-Luc Bonniol. *La Couleur comme maléfice; Une illustration créole de la généalogie des Blancs et des Noirs*, Paris, Albin Michel, 1992

[19] Ransome Y (2020). Religion, spirituality, and health: new considerations for epidemiology. Am J Epidemiol.

[20] Glanz K, Rimer BK, Viswanath K "V." (Eds.). (2015). Theory, research, and practice in health behavior. In K. Glanz, B. K. Rimer, & K. "V." Viswanath (Eds.), Health behavior: Theory, research, and practice (5th ed., pp. 23–41). Jossey-Bass/Wiley.

[21] Bigot R, Croutte P, Daudey E. La diffusion des technologies de L’information et de la communication dans la société française (2013). Collection des Rapports; 2013. p. 297. https://www.credoc.fr/publications/la-diffusion-des-technologies-de-linformation-et-de-la-communication-dans-la-societe-francaise-2014

[22] Robert M. Groves, Floyd J. Fowler Jr., Mick P. Couper, James M. Lepkowski, Eleanor Singer, Roger Tourangeau. *Survey methodology, 2nd edition*: Wiley; 2009.

[23] Don A. Dillman, Jolene D. Smyth, Leah Melani Christian. P. *Internet, Phone, Mail, and Mixed-Mode Surveys: The Tailored Design Method, 4th Edition*: Wiley; 2014. p. 528. https://www.wiley.com/ennl/Internet,+Phone,+Mail,+and+Mixed+Mode+Surveys:+The+Tailored+Design+Method,+4th+Edition-p-9781118456149. ISBN: 978-1-118-45614-9.

[24] Dillman DA, Smyth JD, Christian LM (2014). Internet, phone, mail, and mixed-mode surveys: the tailored design method.

[25] The American Association for Public Opinion Research. Standard definitions. Final dispositions of case codes and outcome rates for surveys revised 2023. https://aapor.org.

[26] Dépistage et prévention du cancer du col de l’utérus 2019 [Haute Autorité de Santé (HAS). Available from: https://www.has-sante.fr/jcms/c_1623735/fr/depistage-et-prevention-du-cancer-du-col-de-l-uterus.

[27] Debussche X, Lenclume V, Balcou-Debussche M, Alakian D, Sokolowsky C, Ballet D (2018). Characterisation of health literacy strengths and weaknesses among people at metabolic and cardiovascular risk: validity testing of the health literacy questionnaire. SAGE Open Med.

[28] Karvonen S, Moisio P, Vepsalainen K, Ollonqvist J (2021). Assessing health gradient with different equivalence scales for household income - a sensitivity analysis. SSM Popul Health.

[29] Krumpal I (2011). Determinants of social desirability bias in sensitive surveys: a literature review. Qual Quant.

[30] Baromètre cancer 2015. Questionnaire. Institut national du cancer, Santé publique France. Saint-Maurice: Santé publique France; 2018. p. 26. https://www.santepubliquefrance.fr.

[31] Estaquio C, Richard JB, Léon C, Arwidson P, Nabi H. Baromètre cancer 2015. Gouvernance et méthodologie de l’enquête. Saint-Maurice: Santé publique France; 2018. p. 10. https://www.santepubliquefrance.fr.

[32] Fenton L, Minton J, Ramsay J, Kaye-Bardgett M, Fischbacher C, Wyper GMA (2019). Recent adverse mortality trends in Scotland: comparison with other high-income countries. BMJ Open.

[33] McCartney G, Hearty W, Arnot J, Popham F, Cumbers A, McMaster R (2019). Impact of political economy on population health: a systematic review of reviews. Am J Public Health.

[34] Mackenbach JP (2017). Persistence of social inequalities in modern welfare states: explanation of a paradox. Scand J Public Health.

[35] Bryere J, Tron L, Menvielle G, Launoy G, French Network of Cancer R (2019). The respective parts of incidence and lethality in socioeconomic differences in cancer mortality. An analysis of the French network Cancer registries (FRANCIM) data. Int J Equity Health.

[36] Moriceau G, Bourmaud A, Tinquaut F, Oriol M, Jacquin JP, Fournel P (2016). Social inequalities and cancer: can the European deprivation index predict patients’ difficulties in health care access? A pilot study. Oncotarget.

[37] Merletti F, Galassi C, Spadea T (2011). The socioeconomic determinants of cancer. Environ Health.

[38] IARC. Cancer Screening in the European Union (2017), Report on the implementation of the Council Recommendation on cancer screening. 2017. https://health.ec.europa.eu/system/files/2017-05/2017_cancerscreening_2ndreportimplementation_en_0.pdf

[39] Mittal A, Neibart SS, Kulkarni A, Anderson T, Hudson SV, Beer NL (2023). Barriers and facilitators to effective cervical cancer screening in Belize: a qualitative analysis. Cancer Causes Control.

[40] Vega Crespo B, Neira VA, Ortiz Segarra J, Andrade A, Guerra G, Ortiz S (2022). Barriers and facilitators to cervical cancer screening among under-screened women in Cuenca, Ecuador: the perspectives of women and health professionals. BMC Public Health.

[41] Jonzo A. « Grâce à la dynamique de l’emploi, le chômage baisse. Enquête emploi 2021 à La Réunion.», Insee Flash La Réunion n° 232, aout 2022. Available from: https://www.insee.fr/fr/statistiques/6483410#documentation.

[42] Grangé C. « Le taux de pauvreté reste stable en 2018 à La Réunion » - Insee Flash Réunion No 194- Janvier 2021. Available from: https://www.insee.fr/fr/statistiques/5016838#:~:text=En%202018%2C%2039%20%25%20des%20Réunionnais%20(332%20500%20personnes),(UC%2C%20figure%201).

[43] Logan L, McIlfatrick S (2011). Exploring women’s knowledge, experiences and perceptions of cervical cancer screening in an area of social deprivation. Eur J Cancer Care (Engl).

[44] Galindo JF, Formigari GM, Zeferino LC, Carvalho CF, Ursini EL, Vale DB (2023). Social determinants influencing cervical cancer diagnosis: an ecological study. Int J Equity Health.

[45] Narumoto K, Miyazaki K, Inoue M, Kaneko M, Okada T, Sugimura M (2022). Investigating women’s health issues and help-seeking intentions in primary care in Japan: a cross-sectional study. BMC Prim Care.

[46] Krause L, Dini L, Prutz F (2020). Barriers for women aged 50 years and older to accessing health care in Germany. J Health Monit.

[47] Labeit AM, Peinemann F (2017). Determinants of a GP visit and cervical cancer screening examination in Great Britain. PLoS One.

[48] Quersin F, Serman F, Favre J, Rochoy M, Descamps A, Gers E (2022). Participation rate in cervical cancer screening in general practice related to the proximity of gynecology care facilities: a 3 year follow-up cohort study. Front Public Health.

[49] The HLS19 Consortium of the WHO Action Network M-POHL (2021). International report on the methodology, results, and recommendations of the European Health Literacy Population Survey 2019–2021 (HLS19) of M-POHL.

[50] Baccolini V, Isonne C, Salerno C, Giffi M, Migliara G, Mazzalai E (2022). The association between adherence to cancer screening programs and health literacy: a systematic review and meta-analysis. Prev Med.

[51] DREES. Études et Résultats. mai 2023. n° 1269. Une personne sur dix éprouve des difficultés de compréhension de l’information médicale. https://drees.solidarites-sante.gouv.fr/sites/default/files/2023-06/ER1269.pdf

[52] Australian Bureau of Statistics. National health survey: health literacy. Canberra: ABS. 2018. Available from: https://www.abs.gov.au/statistics/health/health-conditions-and-risks/national-health-survey-health-literacy/latest-release. Cited 2023 July 5.

[53] Debussche X, Corbeau C, Caroupin J, Fassier M, Ballet D, Balcou-Debussche M (2017). Littératie en santé et précarité: optimiser l’accès à l’information et aux services en santé. L’expérience de Solidarité Diabète. Médecine Des Maladies Métaboliques.

[54] Sand FL, Urbute A, Ring LL, Kjaer AK, Belmonte F, Kjaer SK (2023). The influence of overweight and obesity on participation in cervical cancer screening: a systematic review and meta-analysis. Prev Med.

[55] Hamers FF, Plaine J, Assogba F (2019). Baromètre DOM de Santé Publique France 2014. Dépistage du cancer du col de l’uterus.

[56] Lin H, Chang C, Liu Z, Tan H (2020). The effect of the presence of children on adult smoking behaviour: empirical evidence based on China family panel studies. BMC Public Health.

