# Supplementary material for: Socioeconomic and cultural factors associated with pap smear screening among French women living in Réunion Island
Source: BMC Public Health. 2024 Apr 23;24:1125. doi: 10.1186/s12889-024-18633-4 (PMC11041037; doi:10.1186/s12889-024-18633-4)
Supplement: Supplementary file 1 — Supplementary Material 1. [file 12889_2024_18633_MOESM1_ESM.docx]

**Supplementary file 1: Map of Reunion Island**


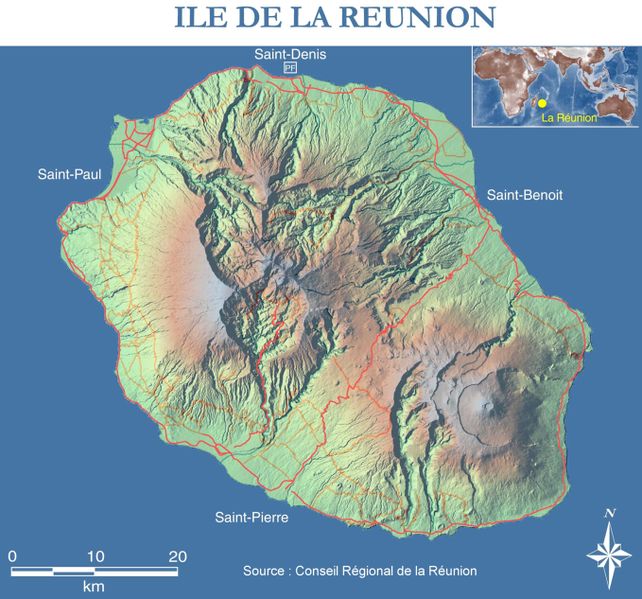


Original file (2,713 × 2,532 pixels, file size : 733 kB, MIME type : image/jpeg)

Description : Map of Reunion Island Date: January 2006 Author: *Conseil Regional de la Reunion License*
