# Supplementary material for: Socioeconomic and cultural factors associated with pap smear screening among French women living in Réunion Island
Source: BMC Public Health. 2024 Apr 23;24:1125. doi: 10.1186/s12889-024-18633-4 (PMC11041037; doi:10.1186/s12889-024-18633-4)
Supplement: Supplementary file 2 — Supplementary Material 2. [file 12889_2024_18633_MOESM2_ESM.docx]

**Supplementary files 2: Data collection design.**

The FOSFORE project was based on a cross-sectional and telephone household survey.

**Data collection.**

Participants were selected by the random dialing system (RDD) with the corresponding area codes (mobile or landline). The questionnaire was administered to participants through a computer-assisted telephone interviewing (CATI system).

**Household member selection.**

For each positive call (one person answered the call men or women):

1. the eligibility of the telephone number has been verified (number associated with a private local domestic unit: exclusion of all telephone numbers dedicated to public institutions, professional services, commercial shop...

2. then, the household unit eligibility has been also verified: at least one on household member is a woman aged 25 to 65.

In the case of several women who could have participated in the survey, automatic random computer selection was systematically made based on age (family rank) to choose the one that was included.

In a second step, at the first direct telephone contact with the selected women, the personal eligibility criteria were verified (voluntary participation, confirmation of age and exclusion of women with a history of uterine surgery).

**Telephone interview**.

For each volunteer, a telephone questionnaire was administered during a specific session (telephone appointment) considering the respondent’s personal availability and preferences.

Data collection ceased when the defined number of complete questionnaires collected was reached.

**Sample Size.**

The probabilistic sampling design of the frame was based on a quota strategy (long formula N = 1000) considering the survey power rules calculation and available financial funds.

Specifically, Dillman et al. showed that a sample of about 1,000 people significantly reduced margin error.

For example, if we consider a split of 80/20 for a closed question encoding Dichotomous question into a binary variable (such as a question with two response-items: updated and not updated in the selection practices), the expected margin error is negligible (at 95% confidence level for a given level of accuracy less than 3 percentage points).

They showed that in this case the minimum required sample size should be 683 individuals is enough to extend outcomes to a targeted population of 1,000,000 to 1,000,000,000 individuals.

| **Response rates** | N |
| --- | --- |
| RDD telephone numbers generation | 29 624 |
| Positive Contacts (someone answer to the call) | 4 643 |
| Exclusion at the first positive contact (ineligible household unit) | 2 645 |
| Eligible Household Unit | 1998 |
| Refusal from the Household member who answered to the first call | 716 |
| Women included after agreement of the household member who have answered to the first call | 1 282 |
| Selected woman refusal (not voluntary to participate) | 8 |
| Medical exclusion (have an history of uterine surgery) | 87 |
| Included woman who did fully answered to the questionnaire (stopped it during the interview) | 191 |
| *Response rate based on full eligibility of women* | *52,3%* |
| *Response rate based on telephonic contact* | *21,5%* |

References:

- Don A. Dillman, Jolene D. Smyth, Leah Melani Christian Internet, Phone, Mail, and Mixed-Mode Surveys: The Tailored Design Method, Wiley; 4e édition (6 août 2014), Hoboken, New Jersey (USA)
- The American Association for Public Opinion Research. Standard Definitions. Final Dispositions of Case Codes and Outcome Rates for Surveys Revised 2023. (https://aapor.org)
