# Supplementary material for: Socioeconomic and cultural factors associated with pap smear screening among French women living in Réunion Island
Source: BMC Public Health. 2024 Apr 23;24:1125. doi: 10.1186/s12889-024-18633-4 (PMC11041037; doi:10.1186/s12889-024-18633-4)
Supplement: Supplementary file 3 — Supplementary Material 3. [file 12889_2024_18633_MOESM3_ESM.docx]

Supplementary files 3: Comparison of the survey population to the Réunion population

|  |  | Crude sample (%) | General population of Reunion Island  INSEE 2013 |
| --- | --- | --- | --- |
| Age (years) | 25-34 | 20.3 | 24.9 |
|  | 35-44 | 23.4 | 27.5 |
|  | 45-54 | 30.5 | 27.4 |
|  | 55-65 | 25.8 | 20.2 |
| Level of education | Higher Education Diplomas | 29.3 | 21.2 |
|  | Baccalaureat | 17.1 | 15.8 |
|  | Cap, Bep | 20.6 | 16.4 |
|  | No Diploma Or Brevet, BEPC | 31.6 | 46.6 |
| Professional situation | Active | 48.3 | 45.6 |
|  | Unemployment | 19.6 | 24.9 |
|  | Other | 32.1 | 29.5 |
| Religion | Christian | 69.7 | 69.6 |
|  | Muslim | 3.5 | 8.6 |
|  | Hindu | 4.8 | 14.8 |
|  | Others | 19.2 | 19 |
| migrants |  |  |  |
|  |  |  |  |
| income families | > 1500€ | 29.8 | 50 |
|  | <1500 € | 62 | 50 |

The French National Institute for Statistics and Economic Studies (INSEE) is dedicated to the production, analysis, and publication of official statistics in France.

We have compared our raw sample with INSEE 2013 data for age, level of education and professional status (1).

Regarding religion and migration, INSEE produces study results at the request of the departments. These factors are not collected exhaustively but based on declarative data from population surveys. 2 publications in 2022 enabled us to compare our gross sample with the population of Reunion Island for religion and migration.

- In 2020, 81% of 18–79-year-olds said they had a religion in Reunion. Among the religions most cited in this study, 86% of believers belong to Christianity, 12% to Hinduism and 7% to Islam (2).
- In Réunion in 2018, 21,000 people were immigrants (i.e. foreign-born abroad), representing 2.5% of the population. Nearly six out of ten of them, or 12,000 people, have French nationality: they have acquired it since their arrival in France. The number of foreign nationals living on Réunion is 10,900, or 1.3% of the island's population. In total, 2.7% of the population residing on Réunion is immigrant and/or of foreign nationality. The proportion of immigrants and foreigners on the island is lower than at national level (3).

For the income of Reunion's population, the "*Budget de famille 2017*" survey conducted by INSEE showed that half of Reunion's people have an income below 1,380 € per month. For our study we have 30% of people with an income above €1,500 and 69% with an income below (4)

**References**

1. S. Ajir. « Bilan démographique 2013 - La croissance de la population ralentit ». Insee Flash Reunion. 2015;n°33.

2. "Panorama des évolutions de la société réunionnaise de 2010 à 2020". **Insee Analyses La Réunion**. n°79. Decembre 2022 [Available from: <https://www.insee.fr/fr/statistiques/6676745#encadre2>.

3. "La part des natifs dans la population diminue, mais demeure élevée". **Insee Analyses La Réunion**. n°74. Octobre 2022 [Available from: <https://www.insee.fr/fr/statistiques/fichier/6536241/re_ina_74.pdf>.

4. INSEE. L'essentiel sur… La Réunion. 29/02/2024 [Available from: <https://www.insee.fr/fr/statistiques/4482473#graphique-figure2_radio1>.
